# An attention base U-net for parotid tumor autosegmentation

**DOI:** 10.3389/fonc.2022.1028382

**Published:** 2022-11-24

**Authors:** Xianwu Xia, Jiazhou Wang, Sheng Liang, Fangfang Ye, Min-Ming Tian, Weigang Hu, Leiming Xu

**Affiliations:** ^1^ The Second Affiliated Hospital, School of Medicine, Zhejiang University, Hangzhou, Zhejiang, China; ^2^ Department of Oncology Intervention, The Affiliated Municipal Hospital of Taizhou University, Taizhou, China; ^3^ Department of Radiation Oncology, Fudan University Shanghai Cancer Center, Shanghai, China; ^4^ Department of Oncology, Shanghai Medical College, Fudan University, Shanghai, China; ^5^ Department of Oncology Intervention, Jiangxi University of Traditional Chinese Medicine, Nanchang, Jiangxi, China

**Keywords:** parotid, auto-segmentation, artificial intelligence (AI), neoplasms, diagnosis

## Abstract

A parotid neoplasm is an uncommon condition that only accounts for less than 3% of all head and neck cancers, and they make up less than 0.3% of all new cancers diagnosed annually. Due to their nonspecific imaging features and heterogeneous nature, accurate preoperative diagnosis remains a challenge. Automatic parotid tumor segmentation may help physicians evaluate these tumors. Two hundred eighty-five patients diagnosed with benign or malignant parotid tumors were enrolled in this study. Parotid and tumor tissues were segmented by 3 radiologists on T1-weighted (T1w), T2-weighted (T2w) and T1-weighted contrast-enhanced (T1wC) MR images. These images were randomly divided into two datasets, including a training dataset (90%) and an validation dataset (10%). A 10-fold cross-validation was performed to assess the performance. An attention base U-net for parotid tumor autosegmentation was created on the MRI T1w, T2 and T1wC images. The results were evaluated in a separate dataset, and the mean Dice similarity coefficient (DICE) for both parotids was 0.88. The mean DICE for left and right tumors was 0.85 and 0.86, respectively. These results indicate that the performance of this model corresponds with the radiologist’s manual segmentation. In conclusion, an attention base U-net for parotid tumor autosegmentation may assist physicians to evaluate parotid gland tumors.

## Introduction

Parotid tumors are uncommon neoplasms, accounting for less than 3% of all head and neck cancers (1). Unfortunately, a lack of early detection may lead to tumor progression, and nearly 20% of untreated polymorphic adenomas will become malignant tumors (2). In addition, 80% of salivary gland tumors occur in the parotid gland, of which 21% to 64% are malignant (3). Due to the absence of specific imaging findings (parotid tumor may have different appearance in MR images), their heterogeneous clinical nature, accurate diagnosis before surgery remains a challenge (4).

Similar to lung nodule detection, automatic parotid tumor segmentation may facilitate physicians evaluating these parotid tumors. It can be used to inspect the MRI image and highlight the tumor region. At the same time, with the progress of quantitative image analysis technology, we can construct a quantitative imaging model of parotid gland tumors through accurate and consistent automatic segmentation of tumors, which can be used to predict the pathological type and prognosis of the patients (5).

In this study, we developed and assessed an autosegmentation model for parotid tumors that can be used to improve the imaging evaluation of these conditions. This proposed model was also compared to other model architectures. Since we combined three MRI sequences, the value of each MRI sequence was investigated.

## Methods

**Figure 1:**
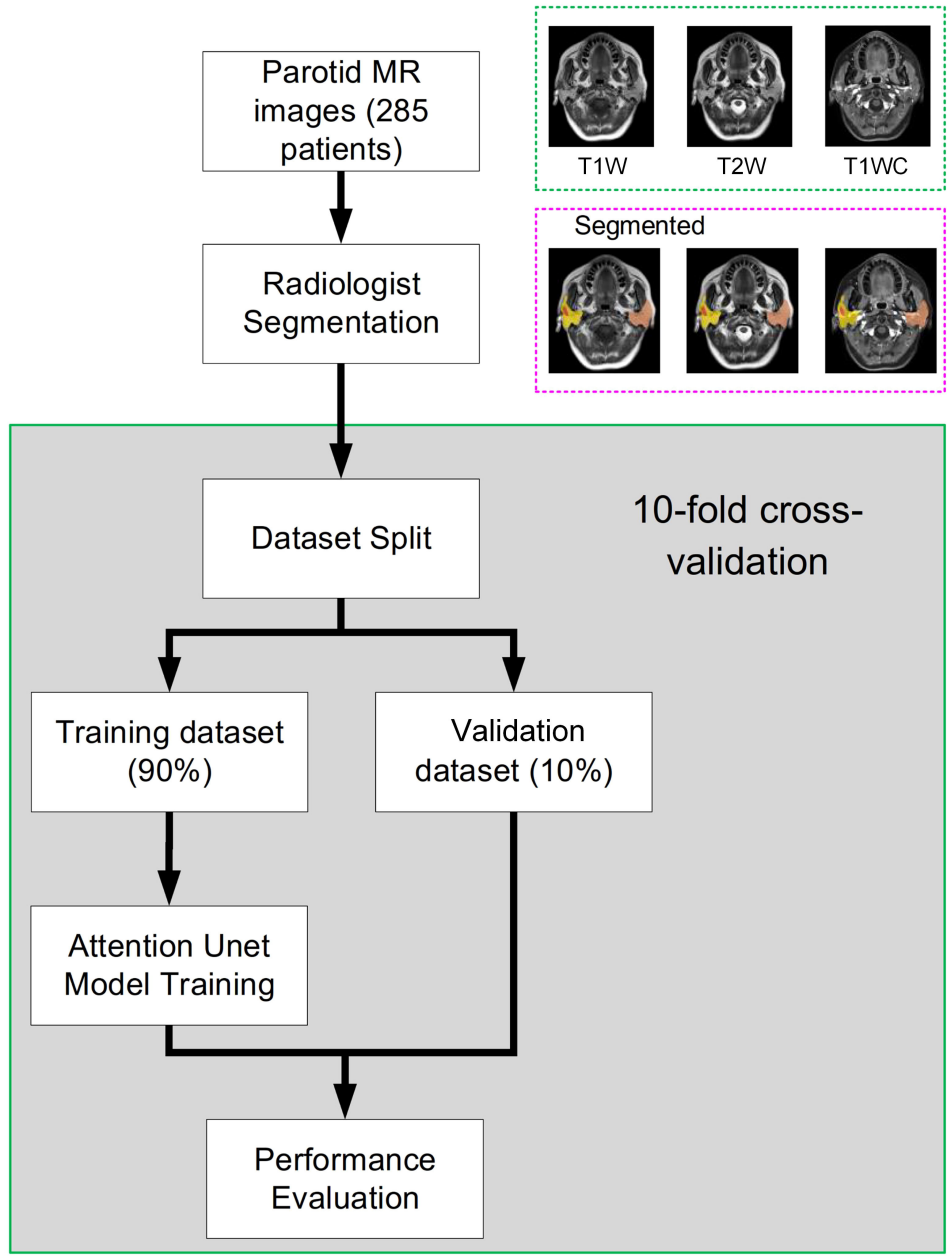
The whole study workflow. The parotid MR images were randomly divided into two datasets, including training and evaluation. Then, the performance was assessed on the validation dataset. Tenfold cross-validation was used to obtain a reliable result.

The study workflow is presented in **Figure 1**. Patient parotid MR images were exported from PACS. Parotid and tumor tissues were segmented by 3 radiologists based on T1-weighted (T1w), T2-weighted (T2w) and T1-weighted contrast-enhanced (T1wC) MR images. A 10-fold cross-validation was performed to assess the segmentation performance. These images were randomly divided into two datasets, including a training dataset (90%) and an validation dataset (10%). The autosegmentation model was trained on the training dataset, and its performance was then tested on the validation dataset. This retrospective study was approved by the Institutional Review Board of Fudan University Shanghai Cancer Center and Taizhou Municipal Hospital, and all methods were performed in accordance with the guidelines and regulations of this ethics board. The Hospital Ethics Committee agreed to the informed consent waiver.

### Patients and MRI image acquisition

Two hundred eighty-five patients diagnosed with benign or malignant parotid tumors from two institutions were enrolled in this study. Among these patients, 185 were male and 100 were female; the mean age of the patients was 52.4 years (range, 21–93 years). These patients were treated from 2014 to 2018. All patients received surgical resection and had a pathology report. The patient characteristics are shown in **Table 1**. All patients received a parotid site MRI scan before treatment. Three MR scanners were used to acquire these images, and details of the image parameters are shown in **Table 2**. The scan parameters were based on our parotid image protocol and were adjusted during scanning based on image quality by the MRI operator.

**Table 1:** Patient characteristics.

		Characteristics
Age		52.4 (21~93) years
Sex	Male	185 (65%)
	Female	100 (35%)
Pathology Type	Warthin tumor	62 (21.5%)
	Pleomorphic adenoma	90 (31.4%)
	Adenocarcinoma	80 (28.0%)
	Basal cell adenoma	6 (2.0%)
	Lymphoma	30 (10.1%)
	Others	20 (7.0%)
Site	Left	127 (44.6%)
	Right	140 (49.1%)
	Both	18 (6.3%)

**Table 2:** MR scan parameters.

		Signa HDxt (GE)	Verio (SIEMENS)	Skyra (SIEMENS)
Patients		218 (76.5%)	34 (11.9%)	33 (11.6%)
T1-weighted	TR (Repetition Time)	280~540 ms	450~620 ms	250~1560 ms
	TE (Echo Time)	8.5~10.4 ms	12~16 ms	2.5~12 ms
T2-weighted	TR (Repetition Time)	2740~3600 ms	2500~5240 ms	2500~5790 ms
	TE (Echo Time)	84~88 ms	78~91 ms	78~83 ms
T1-weighted contrast enhanced	TR (Repetition Time)	175~280 ms	4.1~6.0 ms	3.7~6.0 ms
	TE (Echo Time)	1.8~3.4 ms	1.5~2.5 ms	1.4~2.4 ms
	Contrast Agent	Gadopentetic acid	Gadopentetic acid	Gadopentetic acid
Slice Thickness		5~7 mm	4.5~7.2 mm	4.0~6.0 mm
Pixel size		0.4~0.6 mm	0.65~0.97 mm	0.4~0.85 mm

### Tumor and parotid manual delineation

Parotid tumors were distinguished on axial thin-Section T1w, T2w and T1wC MR images and segmented by three experienced radiologists (>5 years of experience) in MIM (version 6.8.10, Cleveland, US). These three series were registered and fused before segmentation. The radiologists were required to distinguish the pathology type of the parotid tumor before delineation. Each radiologist segmented approximately 90 patients. To make the delineation between different radiologists consistent, all delineations were reviewed by one senior radiologist (more than 10 years’ experience). To improve the performance of the tumor delineation, the parotids were also segmented.

### The attention U-net

**Figure 2:**
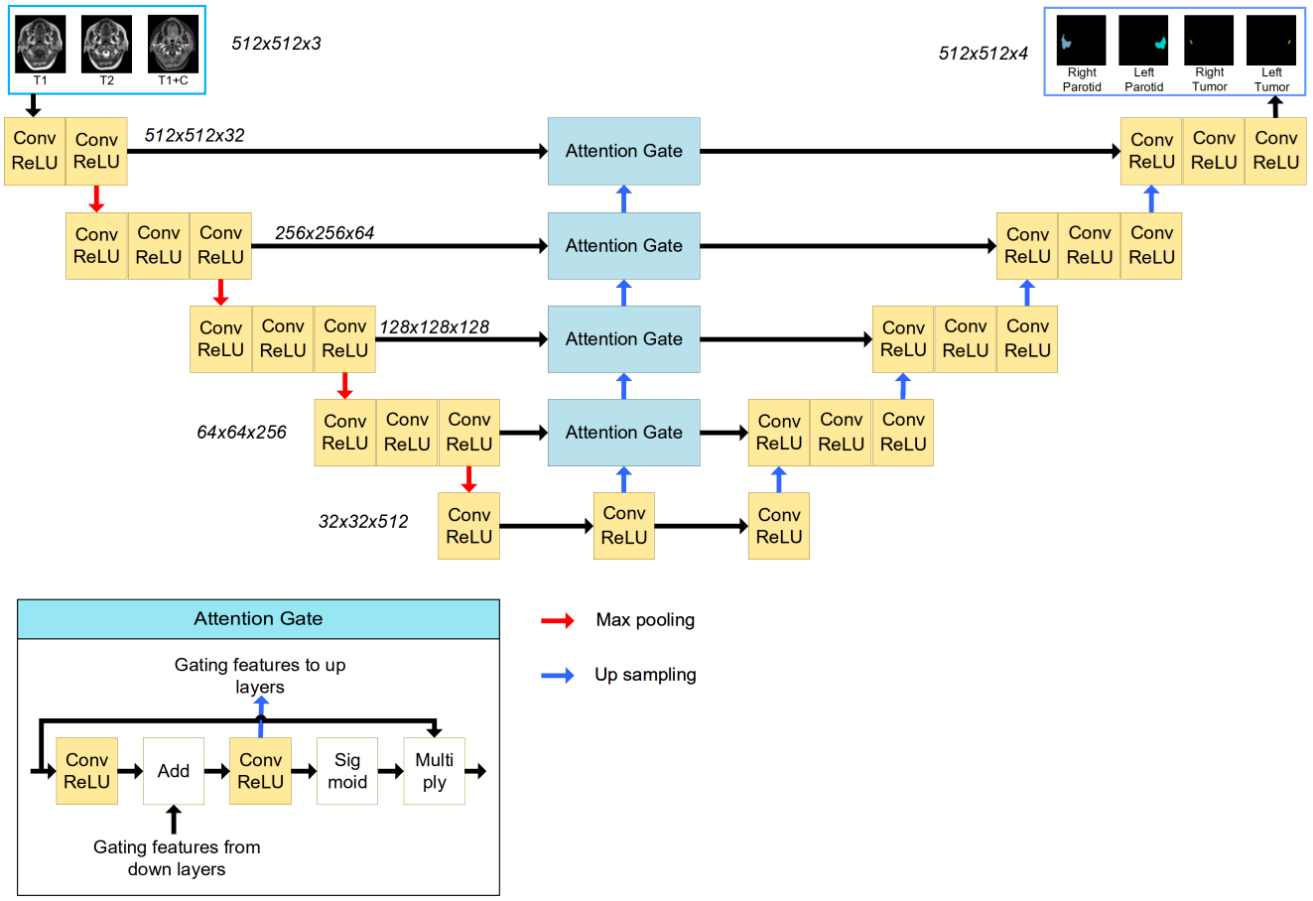
The structure of the attention-based U-Net. The input of the network is three MR images, and the output of the network is the four segmentations. The attention gate structure is shown in the left corner.

A 2D U-Net with an attention module was used in this task. This network was inspired by the application of an attention mechanism to medical image deep learning-based segmentation (6–8). The basic structure of the model is shown in **Figure 2**. The input (512 x 512 x 3) was obtained from MR images. The channels were combined from the T1w, T2w and T1wC sequences. The output (512 x 512 x 4) contained 4 channels for 4 ROIs, including the left parotid, right parotid, left tumor and right tumor. The U-net was constituted by encoder and decoder parts. The encoder part was constituted by 12 convolution blocks and 4 max pooling blocks. The convolution block had a 3x3 convolution layer, batch normalization layer and rectified linear unit (ReLU) layer. The maximum pooling layer was used to downsample the features. Similarly, the decoder part was constituted by many convolution blocks and upsampling blocks. The convolution block was the same as the encoder part, using a 3x3 convolution layer, batch normalization layer and rectified linear unit (ReLU) layer. The skip connection was used to connect the encoder and decoder parts with the same feature map size. An attention gate was placed in these skip connections to improve the segmentation results. Because the slices thickness (4~7.2 mm) was larger than the pixel size (0.4~1mm), MR images were not be resampled to isotropy resolution. And

The tumor and parotid tissues were relatively small compared to the entire image size. The attention mechanism was used to create a model focused on local regions that extracted more relevant features from the feature maps. A mask with pixel values between 0 and 1 was generated by a sigmoid activation function. By multiplying the mask by feature maps, the region of interest remained unchanged, and the rest of the feature map was set to zero.

### Model training

Before input into the model, the gray value of the MR images was centralized to 0.5 and scaled to [0, 1]. No spatial resampling was performed in the preprocessing stage. We used the original pixels, which means that different patients may have different pixel spacings. The loss used in this phase was 1- DICE index. The whole model was trained for 200 epochs with a learning rate of 1e-4, and the optimizer was RMSprop. The training procedure took approximately 20 h to complete on one 2080 ti GPU (Nvidia, Santa Clara, CA). The Python deep learning library pytoch (version 1.5) was applied to establish this autosegmentation system.

Next, a data augmentation method was performed. Two argumentation processes were implemented: gray level disturbance and shape disturbance. For gray disturbance, the gray value of the MR image was multiplied by a random number [0.9~1.1], and a random number [-0.1~0.1] was added. This random number was added to the normalized image. For shape disturbance, MR images and binary contour images were deformed using affine transformation. The augmentation method was the same as that in our previous study (9). Meanwhile, to increase the training samples, we mirrored images (and adjusted for the corresponding left and right labels) with a probability of 0.5.

To investigate the impact of each MRI sequence, 6 models with different image sequence combinations were trained and evaluated, including T1w only, T2w only, T1wC only, T1w+T2w, T1w+T1wC and T2w+T1wC.

### Comparison to other models

Three other models, including DeepLab Version 3 (10), attention U-Net (11) and PSPNet (12), were trained on the same dataset. Some modifications were performed, such as changing the output channels and changing the softmax function to a sigmoid function. The same training hyperparameters were used, and all models converged after 200 epoch iterations.

### Performance evaluation

Four indices were calculated for performance evaluation, including the Dice similarity coefficient (DICE), the Jaccard similarity coefficient (JACCARD), the 95% percentile of Hausdorff distance (HD95) and the average Hausdorff distance (AHD). The DICE and JACCARD are computed by the following:


(1) DICE = 2|A∩B|/(|A|+|B|)

(2) JACCARD = (|A∩B|)/(|A∪B|)

where A represents the volume of the manual segmentation, B represents the volume of the autosegmentation, | · | denotes the volume of truth or predicted ROIs, |A∩B| indicates the volume shared by A and B and |A∪B| represents the total volume of A and B. Larger DICE and JACCARD values indicate more accurate results.

## Results

### Segmentation results

**Figure 3:**
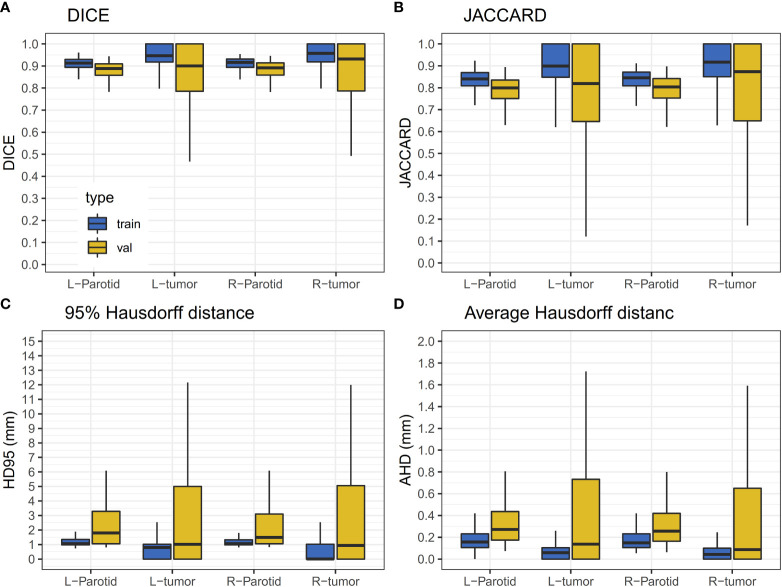
Results of the validation dataset. The horizontal lines indicate the median values. **(A)** The DICE value for the training and validation dataset. **(B)** JACCARD value for the training and validation datasets. **(C)** The HD95 value for the training and validation datasets. **(D)** The AHD value for the training and validation datasets.

A 10-fold cross-validation was used in this study. A total of 256 (90%) patients were used for model training, and 29 (10%) patients were used for model evaluation and performance assessment. Training was converged after 200 epoch iterations. The results of the validation dataset are shown in **Figure 3**. It can be observed that the performance of the validation dataset has a relatively large variation.

**Figure 4:**
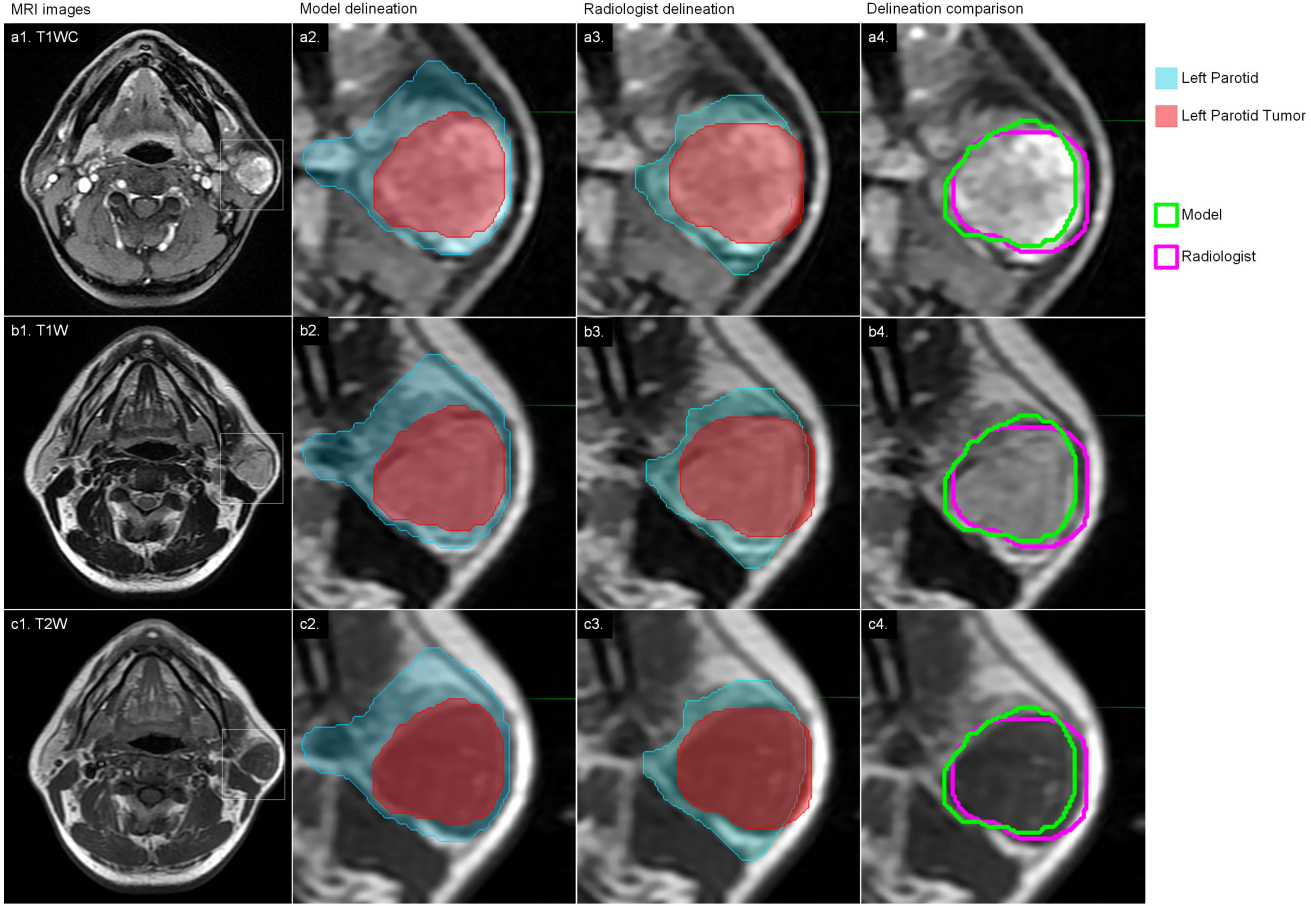
An example of the results. a1, b1 and c1 represent one slice of the MR images; a2, b2 and c2 represent the results of autosegmentation; a3, b3 and c3 represent the results of manual segmentation; a4, b4 and c4 show the comparison of the tumor segmentation.

For the results of the cross-validation, the mean DICE for both parotids was 0.88, and the mean DICE for left and right tumors was 0.85 and 0.86, respectively. The mean JACCARD for left and right parotids was 0.79. The mean JACCARD for left and right tumors was 0.78 and 0.80, respectively. The 95% ranges for left and right parotid DICE were 0.77-0.94 and 0.75-0.95, respectively. The 95% ranges for left and right tumor DICE were 0.37-1.00 and 0.30-1.00, respectively. Detailed values of these results are provided in **Supplementary Table S1**. **Figure 4** demonstrates a result on a left parotid tumor patient.

### Comparison to other models

The performance of three other models, including DeepLab Version 3 (10), attention U-Net (11) and PSPNet (12), is presented in **Table 3**. Since all of the models were trained on the same training dataset, this comparison provides insight into the performance of the proposed model.

**Table 3:** The comparison with other models.

Model	Right parotid		Left parotid		Right tumor		Left tumor	
	DICE	95% CI	DICE	95% CI	DICE	95% CI	DICE	95% CI
DeepLab V3	0.87	[0.65-0.98]	0.85	[0.63-0.95]	0.77	[0.51-0.95]	0.83	[0.45-0.93]
Attention U-Net	0.88	[0.73-0.96]	0.86	[0.71-0.94]	0.84	[0.35-1.00]	0.81	[0.45-1.00]
PSPNet	0.87	[0.72-0.90]	0.85	[0.78-0.89]	0.78	[0.25-1.00]	0.85	[0.38-1.00]
Proposed Model	0.88	[0.75-0.95]	0.88	[0.77-0.94]	0.85	[0.30-1.00]	0.86	[0.37-1.00]

CI, confidence interval.

### The impact of MRI sequences

The performance of models with different MRI sequences is presented in **Table 4**. For parotid gland segmentation, one MRI sequence can achieve segmentation performance similar to that of a combination of three MRI sequences. However, for tumor segmentation, combining three image sequences can provide the best performance. Among the three MRI sequences, T1w performed better than the other two.

**Table 4:** The comparison between different MRI sequences.

MRI Sequences	Right parotid		Left parotid		Right tumor		Left tumor	
	DICE	95% CI	DICE	95% CI	DICE	95% CI	DICE	95% CI
T1w	0.88	[0.74-0.95]	0.86	[0.74-0.92]	0.82	[0.30-1.00]	0.81	[0.37-1.00]
T1wC	0.84	[0.73-0.92]	0.82	[0.69-0.90]	0.71	[0.14-1.00]	0.73	[0.12-1.00]
T2w	0.88	[0.74-0.95]	0.88	[0.72-0.93]	0.81	[0.30-1.00]	0.79	[0.31-1.00]
T1w+T1wC	0.88	[0.75-0.94]	0.85	[0.75-0.92]	0.78	[0.30-1.00]	0.84	[0.53-1.00]
T1w+T2w	0.88	[0.74-0.95]	0.87	[0.75-0.93]	0.84	[0.30-1.00]	0.83	[0.43-0.94]
T1wC+T2w	0.88	[0.75-0.93]	0.85	[0.76-0.94]	0.75	[0.20-1.00]	0.78	[0.30-0.95]
T1w+T1wC+T2w Proposed	0.88	[0.75-0.95]	0.88	[0.77-0.94]	0.85	[0.30-1.00]	0.86	[0.37-1.00]

CI, confidence interval.

## Discussion

In this study, we implemented an attention base U-net for parotid tumor autosegmentation on MRI T1w, T2w and T1wC images. For a rare tumor, the entire dataset was relatively large, including 285 patients, and multiple MRI scanners were used for image acquisition. All whole images were acquired over the course of 4 years with many adjustments to the scan parameters. We believe these images are representative of most parotid tumor MRI scenarios.

An attention mechanism was applied to optimize the extracted spatial information of the feature maps in our study (13). Here, we used a mask with pixel values between 0 and 1 that was generated by transformation, and then feature maps were multiplied by the mask. The region of interest remained unchanged, and the rest of the feature map was set to zero because the regions of the parotid and tumor tissues were relatively small compared to the other organs. This will facilitate model training to focus on critical regions and provide improved results. Compared to the original attention U-Net, our proposed model extracts the gate feature from the bottom of the network. This architecture may help the network focus consistently on only a small region. For hyper-parameters tuning, the major parameters were learning rate. We have use 3 different learn rate (1e-2, 1e-3 and 1e-4), the results showed that 1e-4 can provide the stable results (**Figure S1**).

There are some differences in the difficulty of organs and tumors delineating. Organ delineation is a relatively simple task. Compare to other’s study, our research on the performance of parotid gland segmentation is similar (DICE = 0.88) (14, 15). Few studies have reported using MR imaging for parotid gland autosegmentation. Kieselmann et al. performed atlas-based autosegmentation for parotids (14). The DICE values for Kieselmann’s study were 0.83 and 0.84 for the left and right parotid, respectively. Nuo et al. used deep learning technology on a low-field MR segment of the parotid gland and found that the best performance was 0.85 (15). Compared with these studies, our data were delineated by radiologists with the same protocol on both the training and validation data. The data consistency was relatively good.

**Figure 5:**
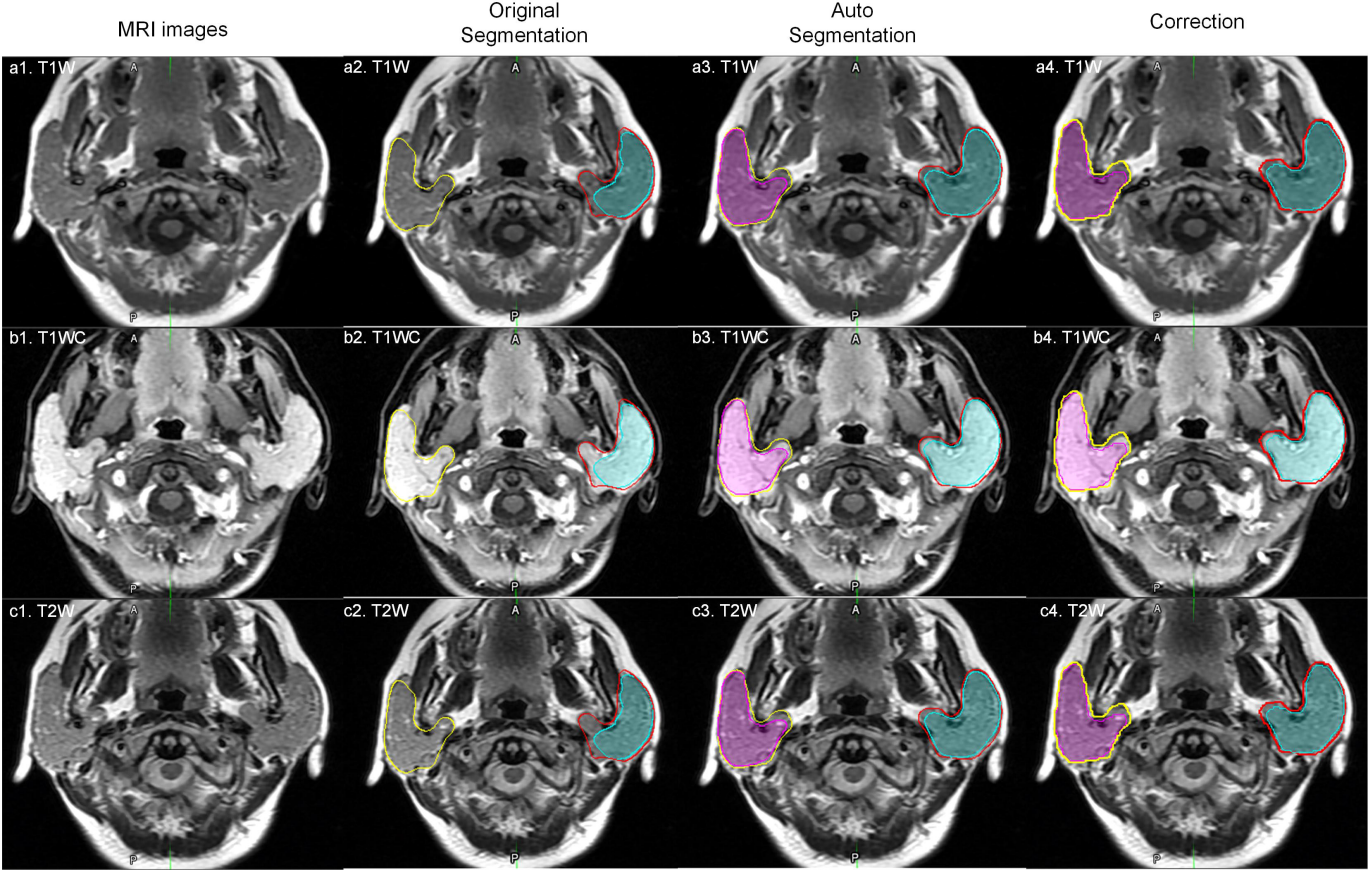
An outlier example. The yellow and red lines represent the right and left parotid. The pink and cyan colored filling represents the right and left tumor. a1, b1 and c1 represent one slice of MR images; a2, b2 and c2 represent original manual segmentation. The right tumor was not delineated correctly; a3, b3 and c3 represent the results of autosegmentation; a4, b4 and c4 represent the corrected segmentation by manual delineation by physicians.

Parotid tumor delineation is a relatively difficult task. The main problem is the lack of training samples and the lack of consistent delineation standards (16). Parotid tumor delineation is challenging in medical image segmentation due to the infrequency of this disease, which physician may not have enough experience to precisely delineate the tumor. Even after carefully reviewed the manual segmentation, there still exist some uncertainty in the manual delineation. **Figure 5** shows a patient with a DICE of 0.127 for a right tumor. After carefully checking the data and reviewing this patient’s history, we found that the delineation in training dataset only segmented part of the tumor, while this patient exhibited a bilateral diffuse MALT (mucosa-associated lymphoid tissue, mucosa-associated lymphoid tissue) lesion. Given this, our model correctly marked the entire tumor, and in this case, the tumor comprised nearly the entire parotid.

There is an overfitting between training and validation. We believe this degree of overfitting is acceptable. While the deviation of performance between different patients still large. For example, the 95% CI of DICE was [0.30-1.00] for of right tumor. This phenomenon indicates that training sample may too small to cover different types of parotid tumors. And the training dataset also may have some uncertainty in delineation.

For the clinical application, because the parotid cancer is a rare cancer, physicians may not have enough experience to assess tumor-infiltrating area. Tumor autosegmentaion may help physicians to do this. Further researches may require to demonstrate the benefit of this model.

There are some limitations to this study. First, we did not validate our model on an external dataset, which might be valuable for providing reliability information. However, because there were 3 MR scanners were used to acquire these images, and the parameters of image protocol were changed during 4 years, using cross validation can precisely estimate the model performance. Second, we combined three images, T1w, T2w and T1wC. For routine diagnostic purposes, some of these images may not be acquired, and a model accounting for missing data may need to be developed in the future.

## Conclusion

An attention base U-net for parotid tumor autosegmentation may assist physicians to evaluate parotid gland tumors.

## Data availability statement

The original contributions presented in the study are included in the article/**Supplementary Material**. Further inquiries can be directed to the corresponding authors.

## Ethics statement

The studies involving human participants were reviewed and approved by Institutional Review Board of Fudan University Shanghai Cancer Center and Taizhou Municipal Hospital. Written informed consent for participation was not required for this study in accordance with the national legislation and the institutional requirements.

## Author contributions

JW, XX, LX, and WH designed study. Data collection: SL, FY, and M-MT. Image segmentation: SL and FY. Data analysis and interpretation XX and JW. Manuscript written – all authors contributed. All authors read and approved final manuscript.

## Funding

The work was supported by the Zhejiang Provincial Health Science and Technology Project. Grant number: 2021KY396.

## Conflict of interest

The authors declare that the research was conducted in the absence of any commercial or financial relationships that could be construed as a potential conflict of interest.

## Publisher’s note

All claims expressed in this article are solely those of the authors and do not necessarily represent those of their affiliated organizations, or those of the publisher, the editors and the reviewers. Any product that may be evaluated in this article, or claim that may be made by its manufacturer, is not guaranteed or endorsed by the publisher.

## Abbreviations

DICE, Dice similarity coefficient.
