# Supplementary material for: An attention base U-net for parotid tumor autosegmentation
Source: Front Oncol. 2022 Nov 24;12:1028382. doi: 10.3389/fonc.2022.1028382 (PMC9730401; doi:10.3389/fonc.2022.1028382)
Supplement: Supplementary file 1 [file DataSheet_1.docx]

supplementary

Table S1. The result of training and validation dataset

| Dataset | ROI | Index | mean | median | 25% | 75% | IQR | 2.5% | 97.5% |
| --- | --- | --- | --- | --- | --- | --- | --- | --- | --- |
| train | R-Parotid | DICE | 0.91 | 0.92 | 0.89 | 0.93 | 0.04 | 0.84 | 0.95 |
| train | R-Parotid | JACCARD | 0.83 | 0.85 | 0.81 | 0.87 | 0.06 | 0.72 | 0.90 |
| train | R-Parotid | HD95 (mm) | 1.38 | 1.06 | 0.98 | 1.33 | 0.35 | 0.79 | 4.37 |
| train | R-Parotid | AHD (mm) | 0.19 | 0.15 | 0.11 | 0.23 | 0.13 | 0.07 | 0.55 |
| train | L-Parotid | DICE | 0.91 | 0.91 | 0.89 | 0.93 | 0.04 | 0.83 | 0.95 |
| train | L-Parotid | JACCARD | 0.83 | 0.84 | 0.81 | 0.87 | 0.06 | 0.71 | 0.90 |
| train | L-Parotid | HD95 (mm) | 1.38 | 1.06 | 0.98 | 1.34 | 0.37 | 0.79 | 4.00 |
| train | L-Parotid | AHD (mm) | 0.23 | 0.16 | 0.11 | 0.23 | 0.13 | 0.07 | 0.49 |
| train | R-tumor | DICE | 0.95 | 0.96 | 0.92 | 1.00 | 0.08 | 0.81 | 1.00 |
| train | R-tumor | JACCARD | 0.91 | 0.92 | 0.85 | 1.00 | 0.15 | 0.68 | 1.00 |
| train | R-tumor | HD95 (mm) | 0.72 | 0.00 | 0.00 | 1.02 | 1.02 | 0.00 | 5.00 |
| train | R-tumor | AHD (mm) | 0.10 | 0.04 | 0.00 | 0.10 | 0.10 | 0.00 | 0.56 |
| train | L-tumor | DICE | 0.95 | 0.95 | 0.92 | 1.00 | 0.08 | 0.80 | 1.00 |
| train | L-tumor | JACCARD | 0.91 | 0.90 | 0.85 | 1.00 | 0.15 | 0.67 | 1.00 |
| train | L-tumor | HD95 (mm) | 0.89 | 0.79 | 0.00 | 1.02 | 1.02 | 0.00 | 5.00 |
| train | L-tumor | AHD (mm) | 0.13 | 0.06 | 0.00 | 0.10 | 0.10 | 0.00 | 0.81 |
| validation | R-Parotid | DICE | 0.88 | 0.89 | 0.86 | 0.91 | 0.06 | 0.75 | 0.94 |
| validation | R-Parotid | JACCARD | 0.79 | 0.80 | 0.75 | 0.84 | 0.09 | 0.60 | 0.88 |
| validation | R-Parotid | HD95 (mm) | 2.46 | 1.49 | 1.06 | 3.10 | 2.04 | 0.90 | 7.24 |
| validation | R-Parotid | AHD (mm) | 0.36 | 0.26 | 0.16 | 0.42 | 0.26 | 0.08 | 1.50 |
| validation | L-Parotid | DICE | 0.88 | 0.89 | 0.86 | 0.91 | 0.05 | 0.77 | 0.94 |
| validation | L-Parotid | JACCARD | 0.79 | 0.80 | 0.75 | 0.84 | 0.08 | 0.63 | 0.88 |
| validation | L-Parotid | HD95 (mm) | 2.77 | 1.80 | 1.06 | 3.29 | 2.23 | 0.90 | 6.08 |
| validation | L-Parotid | AHD (mm) | 0.60 | 0.27 | 0.17 | 0.44 | 0.26 | 0.09 | 1.21 |
| validation | R-tumor | DICE | 0.86 | 0.93 | 0.79 | 1.00 | 0.21 | 0.30 | 1.00 |
| validation | R-tumor | JACCARD | 0.80 | 0.87 | 0.65 | 1.00 | 0.35 | 0.17 | 1.00 |
| validation | R-tumor | HD95 (mm) | 3.45 | 0.94 | 0.00 | 5.06 | 5.06 | 0.00 | 26.17 |
| validation | R-tumor | AHD (mm) | 0.81 | 0.09 | 0.00 | 0.65 | 0.65 | 0.00 | 7.44 |
| validation | L-tumor | DICE | 0.85 | 0.90 | 0.79 | 1.00 | 0.22 | 0.37 | 1.00 |
| validation | L-tumor | JACCARD | 0.78 | 0.82 | 0.65 | 1.00 | 0.35 | 0.23 | 1.00 |
| validation | L-tumor | HD95 (mm) | 3.97 | 1.02 | 0.00 | 5.00 | 5.00 | 0.00 | 26.99 |
| validation | L-tumor | AHD (mm) | 1.04 | 0.14 | 0.00 | 0.73 | 0.73 | 0.00 | 6.99 |

Figure S1. The result of different learning rate


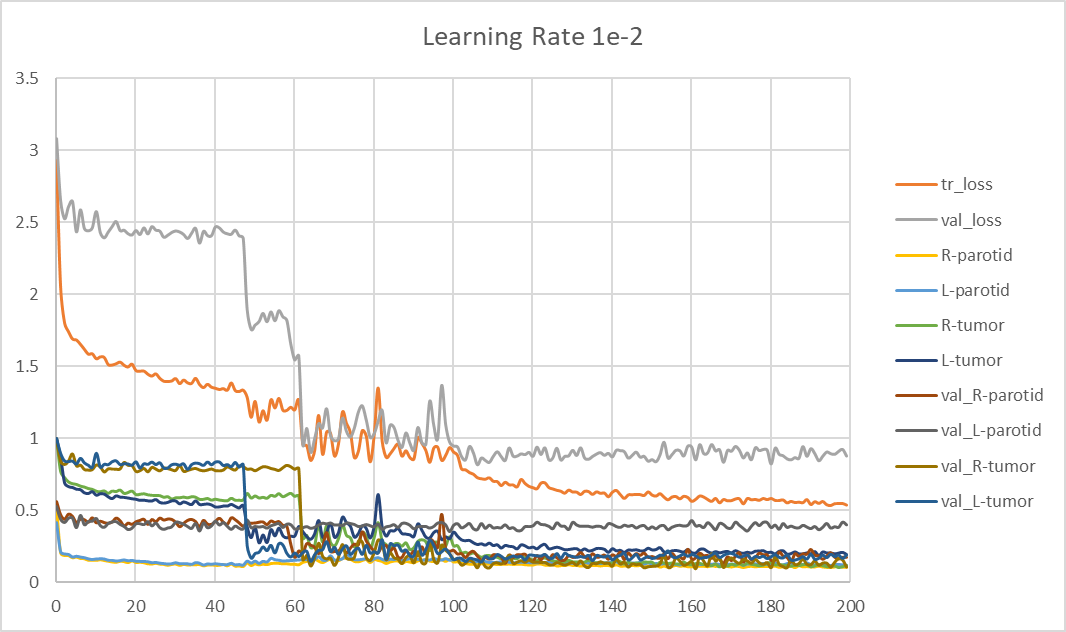

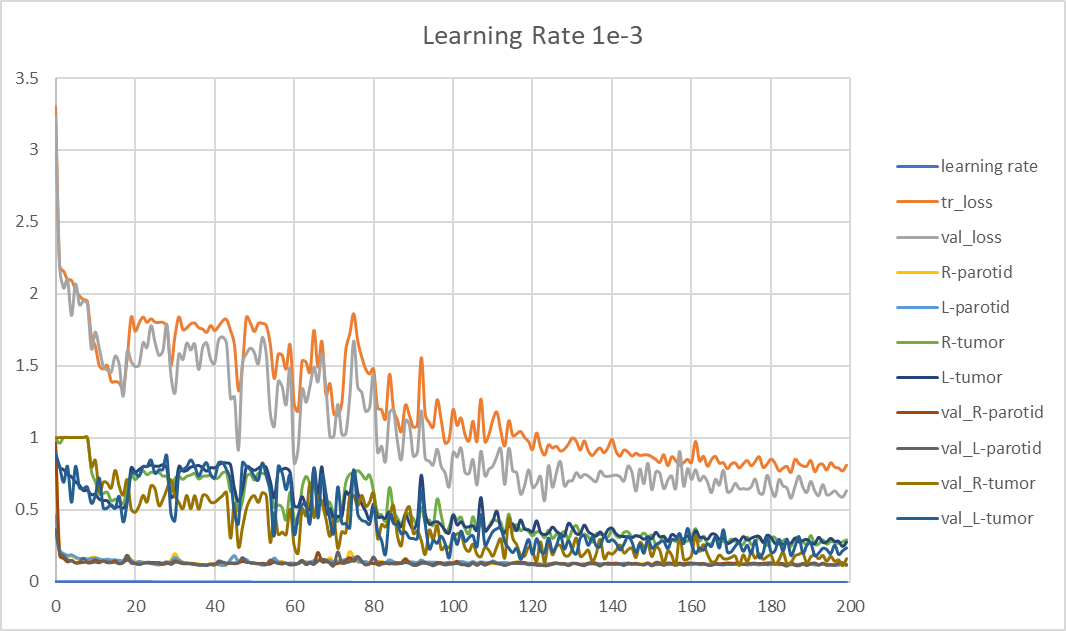

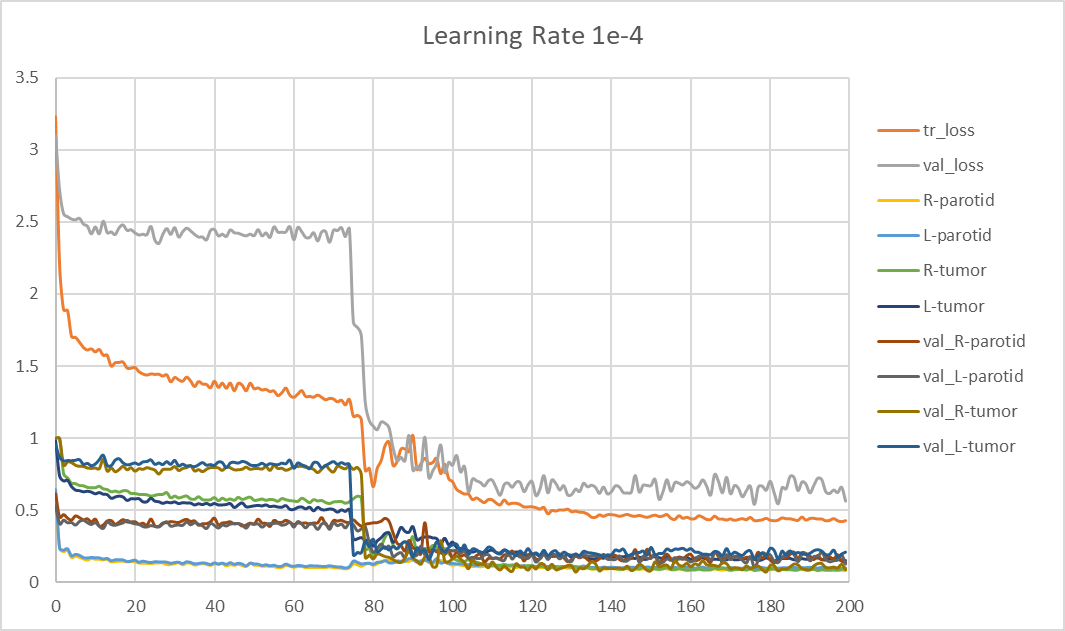


Figure S1. The result of different learning rate. The learning rate for 1^st^ figure was 1e-2; the learning rate for 2^nd^ figure was 1e-3; the learning for 3^rd^ figure was 1e-4.
